# Falling and heaviness: Heaviness judgment for a visual object which users lift up is influenced by the presentation of the object's falling or staying still

**DOI:** 10.3389/fpsyg.2023.1042188

**Published:** 2023-03-28

**Authors:** Yusuke Ujitoko, Seitaro Kaneko, Takumi Yokosaka, Takahiro Kawabe

**Affiliations:** NTT Communication Science Laboratories, Nippon Telegraph and Telephone Corporation, Atsugi, Kanagawa, Japan

**Keywords:** heaviness, visual judgment, falling speed, lifting speed, GUI

## Abstract

When lifting and subsequently releasing a visual object on a screen using a computer mouse, users tend to judge the object to be heavier when the motion speed of the object during lifting is smaller. However it was unclear how the presentation of an object falling after its release influenced the judgment of heaviness. Users generally believe mistakenly that heavier objects fall faster. Based on the previous report of this misbelief, we briefly explored how the falling speed of a visual object after release by a user influenced the judgment of heaviness. The falling speed of the object was systematically modulated by changing gravity in the simulation of the natural falling of the object. Participants judged the object's heaviness after they lifted and subsequently released it. As a result, the participants judged the object to be lighter when the falling speed was zero. However, no significant differences were observed among the conditions with a falling speed greater than zero. It is suggested that for the judgment of heaviness, a vital aspect in the presentation of a falling object after releasing is whether the object falls or not.

## 1. Introduction

When users manipulate a visual object displayed on a screen, the users often feel the property of the object (e.g., heaviness) or the physical property of an interaction between visual objects (e.g., friction) based on the visual features of the object. Among these physical properties, this study focuses on the judgment of the heaviness of the visual object. Previous studies have shown that the heaviness judgment varied with the motion speed of a visual object when lifted up by users in Augmented Reality/Virtual Reality spaces or on touchscreens (Dominjon et al., 2005; Taima et al., 2014; Ban and Ujitoko, 2018; Rietzler et al., 2018; Weser and Proffitt, 2018). Hereafter, we refer to this motion speed during lifting as the “lifting speed.” Ban and Ujitoko asked participants to lift a visual object up to a certain height on touchscreens by using their finger and then release it (Ban and Ujitoko, 2018). To investigate the effect of lifting speed on the judgment of heaviness, Ban and Ujitoko manipulated the ratio of the motion speed of the lifted object on the screen to the movement speed of a participant's finger to drag and lift up the object. We refer to this ratio as “lifting speed ratio.” When the lifting speed ratio was 1, the lifting speed of the object on the display was the same as the movement speed of the participant's finger. When the lifting speed ratio was <1, the lifting speed was less than the movement speed of the participant finger. After the participants lifted and released the object, it fell to the ground. Their participants reported different levels of object's heaviness depending on the lifting speed ratio. Specifically, when the lifting speed ratios of the object were large or small, the object was judged to be light or heavy, respectively. A similar relationship between lifting speed and the judged heaviness was also confirmed in other studies employing different methods of manipulating the object's lifting speed (Dominjon et al., 2005; Taima et al., 2014; Rietzler et al., 2018; Weser and Proffitt, 2018).

There is a possibility that the visual features of an object presented after users lift it can be a cue to the judgment of heaviness. The brain can perceive meaningful events by temporally integrating visual information. The perception of collision events is established by grouping temporally continuous information as a Gestalt (Ryu and Oh, 2018). When two or more object motions are involved, observers can perceive causal events known as Launching effect (Michotte et al., 1964) as well as the mass of each object (Natsoulas, 1960). The kinematic specification of dynamics principle has been proposed as a theory to explain how the human brain inversely infers dynamics from visual kinematics (Runeson and Frykholm, 1983). Although the previous literature has shown the role of temporal integration of motion signals in the formation of visual events, there has been no study investigating whether or how the presentation of an object after users lifted and then released it could alter the heaviness judgment of the object. Although previous studies (Ban and Ujitoko, 2018; Weser and Proffitt, 2018) presented an object falling after participants lifted and then released it, the motion speed of falling was not controlled. Therefore, it was unclear how the motion speed of falling influenced the heaviness judgment. Hereafter, we refer to the motion speed during falling as the “falling speed” although it may actually increase due to gravitational acceleration.

The present study briefly investigated the effect of object's falling speeds on the heaviness judgment. In physics, the falling speed of an object is not dependent on its mass in a situation where air resistance can be ignored. In other words, the falling speeds of objects whose masses are different are identical to each other. Humans cannot perceive the physics of the external world directly and rather often make inaccurate and biased judgments about physical phenomena (Kaiser et al., 1986). In contrast to the physical principle of this mass-speed relationship, users have a misbelief that a heavier object falls faster than a lighter one (Shanon, 1976; Vicovaro, 2014). Recent studies (Vicovaro et al., 2019, 2021) showed that this misbelief was not only related to mental imagery but also was extended to the judgment of actual visual events. They showed a positive relationship between the implied mass of the visual object and the falling speed that was perceived as most natural by observers. There was therefore a probability that if this misbelief could be extended to the heaviness judgment when users watch an object falling after lifting and releasing it, the objects with a smaller or larger falling speed would be judged to be lighter or heavier, respectively.

To investigate the effect of falling speed on the heaviness judgment, we conducted a psychophysical experiment wherein we manipulated the gravitational acceleration applied to a visual object that participants lifted up and then released using a computer mouse. Based on the previous studies (Dominjon et al., 2005; Taima et al., 2014; Ban and Ujitoko, 2018; Rietzler et al., 2018; Weser and Proffitt, 2018), we also manipulated the lifting speed ratio to test the effect of interaction between the lifting speed ratio and the falling speed. This study was the first to test the effect of lifting speed on heaviness judgment when participants manipulated the object with computer mouse.

## 2. Method

### 2.1. Participants

In total, 128 people participated in the experiment. Each age group (the 20, 30, 40, and 50 s) consisted of 16 male and 16 female participants and the mean age was 40.43 (SD: 10.93). To assess the general effect that is applicable to a broader population, we recruited participants from a wide age range. The participants were recruited online by a crowdsourcing research agent in Japan and were paid for their participation. Only people who could participate in the experiment using their own personal computers were recruited and they were unaware of the specific purpose of the experiment. Ethical approval for this study was obtained from the ethics committee at Nippon Telegraph and Telephone Corporation (Approval number: R02-009 by NTT Communication Science Laboratories Ethics Committee). The experiments were conducted according to the principles that have their origin in the Helsinki Declaration. Written informed consent was obtained from all participant in this study.

**Figure 1:**
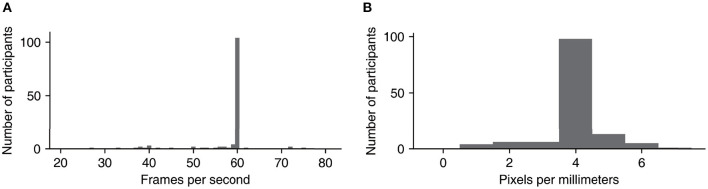
**(A)** Number of participants for each frames per second. **(B)** Number of participants for each pixels per millimeters.

While the total number of participants was 128, we analyzed only data from participants who were tested under specific frames per second (fps) and screen resolution conditions. We recognized that our experimental codes were incomplete insofar as they did not properly address the issue of varying fps and screen resolution conditions arising from differences in the participants' PC environments, and consequently, all participants were not always exposed to visual stimuli having the same spatiotemporal properties. The distribution of fps and screen resolution for all participants is presented in Figures 1A, B, which show that the majority of the participants undertook the experiment in an environment with an fps of 60 and a resolution of 4 pixels per millimeters. By concentrating on the data from participants with these levels of fps and resolution, we were able to exclude the unintended effect of varying fps and screen resolution on the physical falling speed stimuli. Consequently, the data from 78 participants were analyzed.

### 2.2. Stimuli

**Figure 2:**
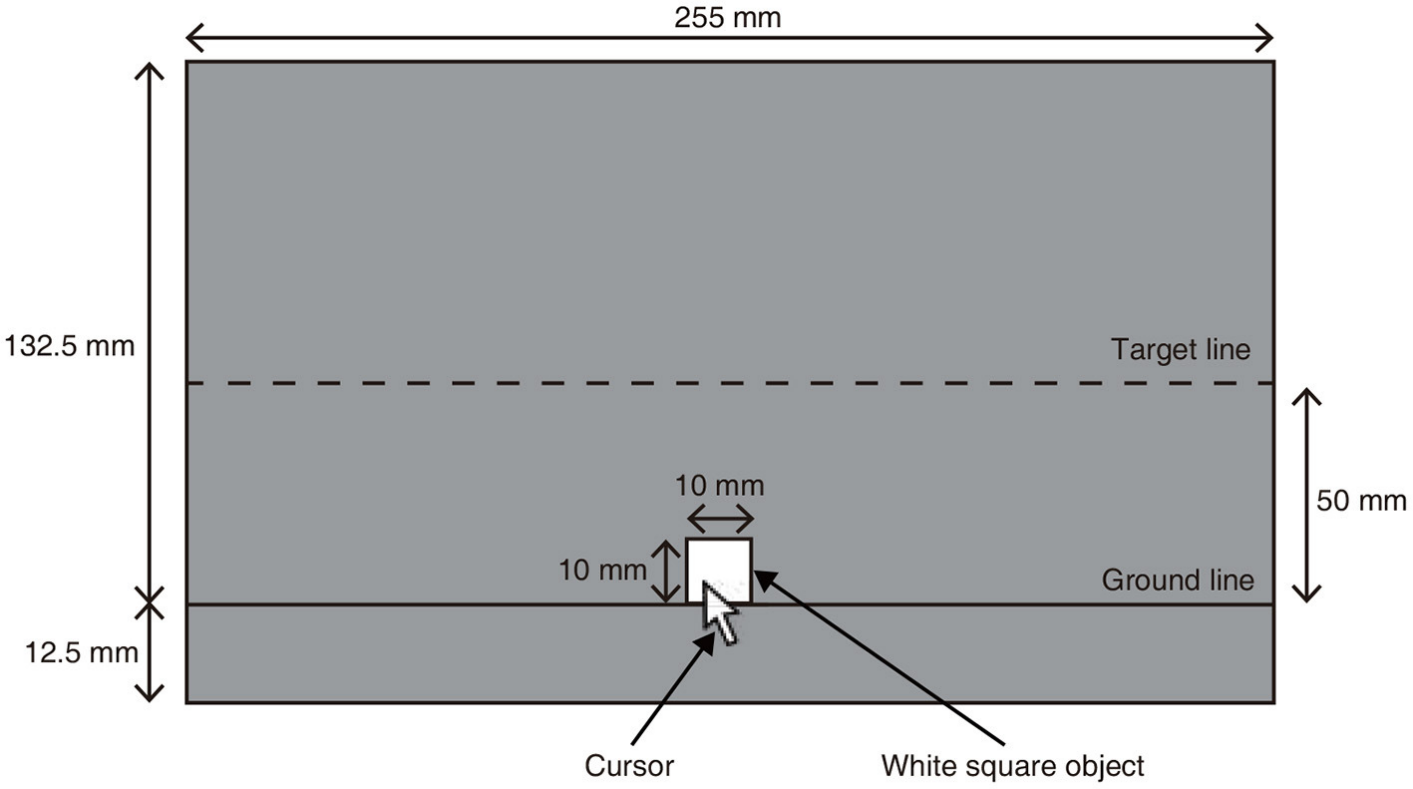
Experimental environment.

In the experiment, participants were instructed to drag a white square object, which was initially placed on a ground line, by using the computer mouse, and to lift it up to a target line (see Figure 2). While lifting, the participants could also move the square object horizontally. When the object reached or passed the target line, participants were instructed to release the object by releasing the button of the mouse in their own timing. After the participant's release, the object fell to the ground line. During the phase of lifting, the speed of the cursor (i.e., the white arrow as shown in Figure 2) was controlled by our experimental system, following the previous study (Ban and Ujitoko, 2018). When the participants were not dragging the object, the speed of the cursor was not controlled by the experimental system and was identical to the default speed of the cursor in the operation system of a participant's computer. When the participants were dragging the object, the cursor speed was controlled to be the default speed multiplied by a lifting speed ratio. For example, when the lifting speed ratio was 0.5, the cursor's speed was half of the default speed of the cursor. There were three conditions of lifting speed ratio (0.25, 0.5, and 1.0).

We also manipulated the falling speed of the object by systematically changing the acceleration due to gravity. Immediately after the participant released the object, the speed of the object was zero. As time passed, the object fell toward the ground line at falling speeds that followed the acceleration due to gravity. The object's vertical position was updated in each frame. In physics, the velocity *v* (that is, speed) of a naturally falling object at time *t* under gravity *g* is described in *v* = *gt*, where air resistance is ignored for simplicity. We simulated a situation in which an object falls from a height of 0.54 m with a gravitational acceleration of 9.8 *m*/*s*^2^ at a distance of 6.53 m from a camera in the simulated world. In our experimental settings, the situation was visualized and presented as a video on the screen. Participants observed the video at a distance of 60 cm from the screen. The falling distance of the object on the screen was 50 mm in height. To investigate the effect of falling speed on the heaviness judgment, we also tested 0.25, 0.5, 2, and 4 times values of the acceleration 9.8*m*/*s*^2^. These levels were chosen so that the difference in the chosen falling speeds was large enough to be visually discriminated by the participants. We also investigated the value of 0 to see how the heaviness judgment was influenced when the object stayed in place after its release. By comparing the judgments made under the conditions of zero and non-zero acceleration, we also checked how the judgment was affected by whether the object fell or stayed in place after its release. Eventually, we configured six conditions of falling speed, which were manipulated by a range of falling accelerations (0, 2.45, 4.9, 9.8, 19.6, and 39.2 *m*/*s*^2^). When the falling acceleration was larger than 0, the object fell to the ground line. When the bottom surface of the falling object made contact with the ground line, the object stopped there. When the falling acceleration was 0, the object stayed at the position of release of the object. The duration of presentation for the object after the release was 3 s. There were thus three conditions of lifting speed ratio and six conditions of the falling speed, giving in total 18 conditions.

### 2.3. Procedure

First, in order to control the distance of observation between the screen and the participants, we instructed the participants to observe the screen from a distance of 60 cm. In addition, in order to control the size of the visual stimulus such as the white object displayed on the monitor regardless of the screen resolution, the screen resolution of the participants was measured according to the method of Li et al. (2020). Specifically, the participants were asked to adjust the size of the rectangle displayed on the screen to be the same as the size of a credit card using the leftward and rightward arrow keys. We controlled the size of the visual stimulus such as the white object displayed during the experiment based on the measured screen pixel size. Next, the participants were presented with written descriptions explaining the purpose of the experiment and specific task contents. After the participants completed reading the descriptions, they started the experiment by pressing the enter key.

On each trial, the participant's task in the experiment was to drag a stationary white object on the ground line to the target line and release the mouse button. There was no instruction about the dragging speed. After releasing the mouse button, the object fell from, or stayed at, the released position according to the falling acceleration in that trial. After the release of the button, the object was presented for 3 s. Then, an answer screen appeared. The answer screen asked the following question: “Please judge the heaviness of the object.” Participants answered to this question using the Visual Analog Scale (VAS) (Crichton, 2001). Participants were instructed to click on a position more to the right on the VAS when they judged the object to be heavier. The VAS has a width of 15 cm. To reduce the bias of the answer, we used the Banded design (Matejka et al., 2016) with two labels (“I don't feel heaviness at all” and “I feel the heaviest feeling I can imagine”) as the VAS design.

The experiment was composed of familiarization and test phases. In the familiarization phase, each participant performed the required operation for six conditions, which were randomly extracted from the 18 conditions. After these had been completed, the test phase started. In the test phase, each participant performed the operation for the 18 conditions once. The presentation order of the 18 conditions in the test phase was pseudo-randomly assigned to each participant.

## 3. Results

### 3.1. Heaviness rating scores varied with lifting and falling speeds

**Figure 3:**
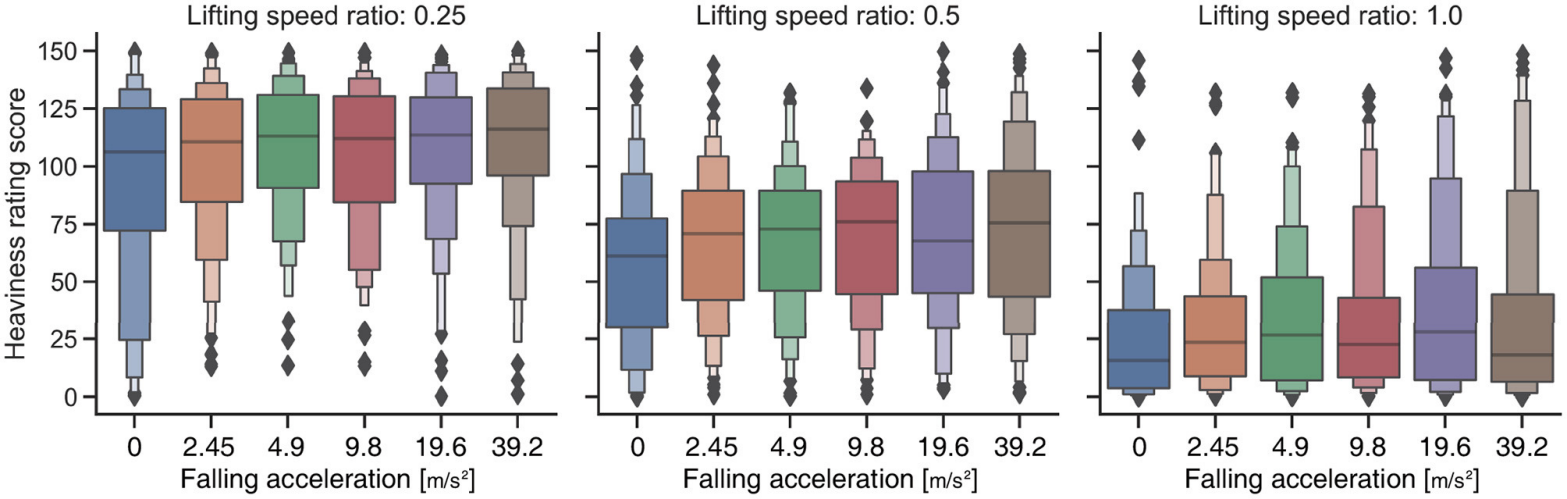
Heaviness rating score for each combination of lifting speed ratio and falling acceleration. The y-axis corresponds to the visual analog scale (VAS) presented to participants. The smallest value of the VAS is 0 (corresponding to “I don't feel heaviness at all”) and the largest value of the VAS is 150 (corresponding to “I feel the heaviest feeling I can imagine”).

The heaviness rating scores for each condition are shown in Figure 3. To clarify how the heaviness rating scores changed with the factors that we manipulated, we conducted an Aligned Rank Transform (ART) (Wobbrock et al., 2011) on the data and then a two-way within-participants ANOVA on the aligned ranks. There were significant main effects of lifting speed ratio [*df* = 2, *F* = 240.0, *p* < 0.001, $\eta_{p}^{2}=0.756$] and falling speed [*df* = 5, *F* = 4.7, *p* < 0.001, $\eta_{p}^{2}=0.058$]. There was no significant interaction effect [*df* = 10, *F* = 1.3, *p* = 0.21, $\eta_{p}^{2}=0.017$].

As a *post-hoc* test, multiple comparisons within the ART paradigm (ART-C) (Elkin et al., 2021) with Bonferroni correction were performed for the main effect of lifting speed ratio. There were significant differences between all pairs of lifting speed ratios (*p* < 0.01). In addition, multiple comparisons were performed for the main effect of the falling speed. There were significant differences only between pairs of 0–4.9, 0–9.8, 0-1-9.6, and 0–39.2 (*p* < 0.05), but there were no significant differences between other pairs (*p* > 0.05).

## 4. Discussion

Our results showed that there were significant effects of both lifting speed ratio and falling speed on the heaviness judgment. In particular, the effect of changes in the lifting speed ratio was greater than the effect of changes in the falling speed. When the lifting speed ratio was smaller, the object was judged to be heavier. The results are consistent with the results of the previous studies demonstrating the strong effect of the lifting speed on the heaviness judgment of an object (Dominjon et al., 2005; Taima et al., 2014; Ban and Ujitoko, 2018; Rietzler et al., 2018).

We found a novel effect of falling events on the judgment of object heaviness. Specifically, we found that the heaviness rating scores under the condition with the falling speed of 0 were significantly smaller than the heaviness rating scores under conditions with the falling speed of 4.9 and more. The results indicate that the judgment of heaviness for an object manipulated by users is influenced not only by lifting speeds but also by whether or not the object falls after its release.

It is unclear why the heaviness rating scores decreased under the condition with the falling speed of 0. We assume that the human cognitive system uses signals in the pre- and post-release phases to judge the object heaviness. An important point is that participants might judge heaviness differently between object being lifted at pre-release phase and object staying at the released position at post-release phase. Specifically, the lifted object was judged to be heavier as the lifting speed was smaller. However, the object staying at the release position was likely judged to be light because it was floating in the air after the release. In addition, the human cognitive system might determine the final estimation of the heaviness on the basis of the heaviness judgment in both pre- and post-release phases. In the previous psychological research, it has been shown that the cognitive system establishes the representation of a single event by considering information presented in a wide temporal range including before and after the event (Bernstein et al., 2004; Miyazaki et al., 2010; Kawabe, 2012; Wu et al., 2012; Liesner et al., 2020). Consistent with the previous studies, the participants might use the heaviness estimation in both pre- and post-release phases and this might result in a lighter judgment of the object's heaviness when the falling speed was zero.

On the other hand, there were no significant differences among conditions with the falling speeds greater than 0 in multiple comparisons. The results do not support the idea that the presentation of falling speed influences the judgment of object heaviness. In our stimuli, falling speeds >0 ranged from 2.45 to 39.2 *m*/*s*^2^. Even this 16 times difference in falling speed did not make significant differences in the heaviness judgment. The result suggests that the falling speed of an object has little effect on the heaviness judgment.

The minor role of falling speed (when more than zero) in the judgment of heaviness might be observed because participants could determine the timing for releasing the object, and thus, as a task they could report the object heaviness by relying mainly on the lifting speed in the pre-release phase. Because a falling object was a natural consequence of the participant's release of the object, the participants probably tended to ignore the appearance of the falling object in the task of judging the object's heaviness. To draw the participant's attention toward a falling object and prevent the participant from ignoring it in the post-release phase, it would be interesting to check the situation in which a lifted object automatically initiates falling after reaching a target line. The automatic initiation of a falling phase would likely capture the participant's attention and prevent the participant from ignoring the object in the falling phase. Another possibility is that a longer distance between the target line and the ground line could make the effect of falling speed more significant. If the distance is longer, the duration of the fall would be longer, and it would allow the participants to make a more focused assessment of the falling speed. It is thus expected that the automatic initiation of the falling phase or a longer falling distance increase the contribution of the falling speed as compared to the conditions adopted in the present study.

The present study could not disentangle the contribution of the falling speed, the falling duration, and the duration of the object lying on the floor to the heaviness judgment. In our stimuli, objects with a smaller gravitational acceleration were involved with a smaller falling speed, a longer falling duration, and a shorter duration of the object's lying on the floor. Although we discussed our results in terms of the falling speeds only, there was a possibility that either or both of the other two factors might be critical, and this is left as an open issue for future studies.

The behavior of the object after landing on the ground line may also be a critical cue to the judgment of the heaviness of the object. Though the object did not bounce at the ground line at the end of the falling phase in our experiment, the behavior of an object after it falls can vary with the type of application. For example, the object may bounce and even interact with other objects at the ground line. It is known that a bouncing trajectory of a falling object influences the perception of the material of the object (Warren et al., 1987; Paulun and Fleming, 2020). Similarly, the bouncing behavior of a falling object may alter the judgment of object heaviness. Clarification of the effect of the bouncing behavior on the judgment of the heaviness of an object should be useful for engineers who want to implement the behavior of objects in applications because it is important for them to know whether the behavior of the object after falling affects the user's judgment of object properties including heaviness.

One might suspect that some participants felt other impressions on the objects. This possibility is consistent with the earlier findings of pseudo-haptics reporting that a decrease in cursor movement speed when using a computer mouse is attributed not only to heaviness but also to other haptic impressions, such as friction (see Table 1 in the review paper Ujitoko and Ban, 2021). On the other hand, since the task set in our experiment was to lift up an object placed on the ground, the word “heaviness” would be more natural for participants than the word “friction.” Further research is warranted to elucidate what contextual information can affect the attribution of the drag difficulty to heaviness, friction, or other factors.

In connection with the above, while our results are considered to be valuable in the field of psychophysical research, in that they have clarified the novel aspects of the heaviness judgment of users, our results would be useful in the field of engineering in situations where the appropriate physical properties of an object are required to be conveyed to the users with computer interfaces as intended by the engineer. In a series of previous studies (Dominjon et al., 2005; Taima et al., 2014; Ban and Ujitoko, 2018; Rietzler et al., 2018; Weser and Proffitt, 2018), it was suggested that the visual features of an object need to be properly designed to convey its physical properties as intended. These previous studies focused only on the visual features during the user's manipulation. In contrast, our study proposes the significant effect of the visual features of an object after the user's manipulation on the judgment of physical properties. This indicates that the engineers need to design the visual features not only during the user's manipulation but also within a wider temporal range (including after manipulation).

There is a possibility that a similar kind of modulation for post-release visual features can alter the judgment of object properties other than heaviness. For example, let us assume the situation wherein users pull an elastic object such as a spring or a soft material presented on the screen by using some input devices such as Spaceball (Lécuyer et al., 2000) or a hand tracker (Kawabe, 2020). It is known that the speed of object deformation during user's pulling can affect the judgment of an object's stiffness. Nevertheless, it is still unclear whether the presentation of the appearance of an elastic object which returns to the initial state might enhance/deteriorate the judgment of the object's stiffness. Our results indicate that it is necessary to closely investigate the influence of visual features presented over a wider temporal range on the judgments of the physical properties of the object for clarifying all characteristics of human perception of material and object physical properties.

## Data availability statement

The raw data supporting the conclusions of this article will be made available by the authors, without undue reservation.

## Ethics statement

The studies involving human participants were reviewed and approved by NTT Communication Science Laboratories Ethics Committee (Approval number: R02-009). The patients/participants provided their written informed consent to participate in this study.

## Author contributions

YU, TY, and TK conceived the experiment. SK and YU implemented the experimental system and analyzed the results. All authors interpreted the data and reviewed the manuscript. All authors contributed to the article and approved the submitted version.

## References

[B1] Ban Y. Ujitoko Y. (2018). Enhancing the pseudo-haptic effect on the touch panel using the virtual string, in 2018 IEEE Haptics Symposium (HAPTICS) (San Francisco, CA: IEEE), 278–283.

[B2] Bernstein D. M. Atance C. Loftus G. R. Meltzoff A. (2004). We saw it all along: visual hindsight bias in children and adults. Psychol. Sci. 15, 264–267. 10.1111/j.0963-7214.2004.00663.x 15043645 PMC3640979

[B3] Crichton N. (2001). Visual analogue scale (vas). J. Clin. Nurs. 10, 706–706.

[B4] Dominjon L. Lecuyer A. Burkhardt J.-M. Richard P. Richir S. (2005). Influence of control/display ratio on the perception of mass of manipulated objects in virtual environments, in IEEE Proceedings. VR 2005. Virtual Reality, 2005 (Bonn: IEEE), 19–25.

[B5] Elkin L. A. Kay M. Higgins J. J. Wobbrock J. O. (2021). An aligned rank transform procedure for multifactor contrast tests, in The 34th Annual ACM Symposium on User Interface Software and Technology (New York, NY), 754–768.

[B6] Kaiser M. K. Jonides J. Alexander J. (1986). Intuitive reasoning about abstract and familiar physics problems. Mem. Cogn. 14, 308–312. 10.3758/BF03202508 3762384

[B7] Kawabe T. (2012). Postdictive modulation of visual orientation. PLoS ONE 7, e32608. 10.1371/journal.pone.0032608 22393421 PMC3290577

[B8] Kawabe T. (2020). Mid-air action contributes to pseudo-haptic stiffness effects. IEEE Trans. Haptics 13, 18–24. 10.1109/TOH.2019.2961883 31880559

[B9] Lécuyer A. Coquillart S. Kheddar A. Richard P. Coiffet P. (2000). Pseudo-haptic feedback: can isometric input devices simulate force feedback? in Proceedings IEEE Virtual Reality 2000 (Cat. No. 00CB37048) (New Brunswick, NJ: IEEE), 83–90.

[B10] Li Q. Joo S. J. Yeatman J. D. Reinecke K. (2020). Controlling for participants' viewing distance in large-scale, psychophysical online experiments using a virtual chinrest. Sci. Rep. 10, 1–11. 10.1038/s41598-019-57204-1 31969579 PMC6976612

[B11] Liesner M. Kirsch W. Kunde W. (2020). The interplay of predictive and postdictive components of experienced selfhood. Conscious Cogn. 77, 102850. 10.1016/j.concog.2019.102850 31731032

[B12] Matejka J. Glueck M. Grossman T. Fitzmaurice G. (2016). The effect of visual appearance on the performance of continuous sliders and visual analogue scales, in Proceedings of the 2016 CHI Conference on Human Factors in Computing Systems (San Jose, CA), 5421–5432.

[B13] Michotte A. Miles T. Miles E. (1964). The perception of causality. Br. J. Philos. Sci. 15, 59.

[B14] Miyazaki M. Hirashima M. Nozaki D. (2010). The “cutaneous rabbit” hopping out of the body. J. Neurosci. 30, 1856–1860. 10.1523/JNEUROSCI.3887-09.2010 20130194 PMC6633980

[B15] Natsoulas T. (1960). Judgments of velocity and weight in a causal situation. Am. J. Psychol. 73, 404–410. 10.2307/1420178 13727951

[B16] Paulun V. C. Fleming R. W. (2020). Visually inferring elasticity from the motion trajectory of bouncing cubes. J. Vis. 20, 6–6. 10.1167/jov.20.6.6 32516356 PMC7416883

[B17] Rietzler M. Geiselhart F. Gugenheimer J. Rukzio E. (2018). Breaking the tracking: Enabling weight perception using perceivable tracking offsets, in Proceedings of the 2018 CHI Conference on Human Factors in Computing Systems (New York, NY: Association for Computing Machinery), 1–12.

[B18] Runeson S. Frykholm G. (1983). Kinematic specification of dynamics as an informational basis for person-and-action perception: expectation, gender recognition, and deceptive intention. J. Exp. Psychol. Gen. 112, 585. 10.1037/0096-3445.112.4.585

[B19] Ryu D. Oh S. (2018). The effect of good continuation on the contact order judgment of causal events. J. Vis. 18, 5–5. 10.1167/18.11.5 30347092

[B20] Shanon B. (1976). Aristotelianism, newtonianism and the physics of the layman. Perception 5, 241–243. 10.1068/p050241 951174

[B21] Taima Y. Ban Y. Narumi T. Tanikawa T. Hirose M. (2014). Controlling fatigue while lifting objects using pseudo-haptics in a mixed reality space, in 2014 IEEE Haptics Symposium (HAPTICS) (Houston, TX: IEEE), 175–180. 10.1109/HAPTICS.2014.6775451

[B22] Ujitoko Y. Ban Y. (2021). Survey of pseudo-haptics: haptic feedback design and application proposals. IEEE Trans. Haptics. 14, 699–711. 10.1109/TOH.2021.3077619 33950845

[B23] Vicovaro M. (2014). Intuitive physics of free fall: an information integration approach to the mass-speed belief. Psicológica 35, 463–477.

[B24] Vicovaro M. Noventa S. Battaglini L. (2019). Intuitive physics of gravitational motion as shown by perceptual judgment and prediction-motion tasks. Acta Psychol. 194, 51–62. 10.1016/j.actpsy.2019.02.001 30743090

[B25] Vicovaro M. Noventa S. Ghiani A. Mena F. Battaglini L. (2021). Evidence of weight-based representations of gravitational motion. J. Exp. Psychol. Hum. Percept. Perform. 47, 1445–1471. 10.1037/xhp0000956 34591519

[B26] Warren Jr W. H. Kim E. E. Husney R. (1987). The way the ball bounces: visual and auditory perception of elasticity and control of the bounce pass. Perception 16, 309–336. 10.1068/p160309 3432028

[B27] Weser V. Proffitt D. R. (2018). Making the visual tangible: substituting lifting speed limits for object weight in VR. Presence 27, 68–79. 10.1162/pres_a_00319

[B28] Wobbrock J. O. Findlater L. Gergle D. Higgins J. J. (2011). The aligned rank transform for nonparametric factorial analyses using only anova procedures, in Proceedings of the SIGCHI Conference on Human Factors in Computing Systems (Vancouver, BC), 143–146.

[B29] Wu D.-A. Shimojo S. Wang S. W. Camerer C. F. (2012). Shared visual attention reduces hindsight bias. Psychol. Sci. 23, 1524–1533. 10.1177/0956797612447817 23085643

